# Understanding the evolution of trust in a participatory health research partnership: A qualitative study

**DOI:** 10.1111/hex.13918

**Published:** 2023-11-29

**Authors:** Meghan Gilfoyle, Anne MacFarlane, Zoe Hughes, Jon Salsberg

**Affiliations:** ^1^ Health Research Institute (HRI) University of Limerick Limerick Ireland; ^2^ Public and Patient Involvement Research Unit University of Limerick Limerick Ireland; ^3^ Care Alliance Ireland Dublin Ireland

**Keywords:** community‐based participatory research, participatory health research, qualitative, social networks, trust

## Abstract

**Introduction:**

Advancements in evaluating the impact of participatory health research (PHR) have been made through comprehensive models like the community‐based participatory research (CBPR) conceptual model, which provides a useful framework for exploring how context and partnership processes can influence health research design and interventions. However, challenges in operationalising aspects of the model limit our understanding and evaluation of the PHR process. Trust is frequently identified as an important component of the CBPR model, which supports the development of key partnership outcomes, such as partnership synergy. However, trust continues to be limited to a binary view (as present or absent), which is problematic given its inherently dynamic and temporal nature.

**Study Aim:**

The aim of this qualitative study is to understand the evolution of trust in the national public and patient involvement (PPI) network in Ireland.

**Setting and Participants:**

Participants from the PPI network (*n* = 15/21) completed a semistructured interview discussing the evolution of trust by reviewing four social network maps derived from a previous longitudinal study.

**Analysis:**

Following Braun and Clarke, we used reflexive thematic analysis, to iteratively develop, analyse and interpret our mediated reflection of the data.

**Results:**

Participants described the evolution of trust as a function of three contextual factors: (1) the set‐up and organisation of the network, (2) how people work together and (3) reflection on the process and outcomes. Their descriptions across these themes seemed to vary depending on partnership type with National Partners and Site Leads having more opportunities to demonstrate trust (e.g., via leadership roles or more resources), compared to Local. Thus, visibility and the opportunity to be visible, depending on the set‐up and organisation of the network and how people work together, seemingly play an important role in the evolution of trust over time. Based on these findings, we provide important questions for reflection across themes that could be considered for future PHR partnerships.

**Discussion:**

Given that the opportunity and visibility to build and maintain trust over time may not be equally available to all partners, it is important to find ways to invest in and commit to equitable relationships as the key to the success (i.e., longevity) of partnerships. We reflect on/offer important implications for those engaging in PHR partnerships and those who fund such research.

**Patient or Public Contribution:**

A Research Advisory Group comprising four research partners (representing academic, service and community organisations) from the PPI Ignite Network provided input and approval for the research objectives of this study as well as previously published work informing this study. Informal consultation occurred with members of this group to discuss findings from this study, assisting with the way findings are presented and described, to be accessible for diverse audiences. Two Research Advisory Group members were involved in the interpretation of the results, and one is a co‐author of this manuscript (Zoe Hughes).

## 1 INTRODUCTION

Participatory health research (PHR) meaningfully and equitably involves people whose lives or work are the subject of the study throughout all stages of the research process.^1^, ^2^, ^3^, ^4^ PHR has grown substantially in recent decades across research communities globally^5^ reflecting its impact ‘on individuals, groups of people, communities of practice, institutions, organisations as well as the relationships and the quality of the research process itself'.^3^ We use PHR as an umbrella term encompassing many terms used in the space of collaborative production (e.g., community‐based participatory research [CBPR], integrated knowledge translation, public and patient involvement [PPI], etc.).

Advancements in evaluating the impact of PHR have been made through comprehensive models like the CBPR conceptual model, which provides a useful framework for exploring how context and partnership processes can influence health research design and interventions.^1^ ^,^^p.^ ^81^ However, challenges in operationalising aspects of the model persist, limiting our understanding of understanding and evaluating the PHR process. Trust is frequently identified as an important component of the CBPR model, which supports the development of key partnership outcomes, such as partnership synergy.^6^, ^7^ Despite its importance, trust continues to be limited to a binary view (as present or absent)^8^, ^9^ which is problematic given its inherently dynamic and temporal nature.^10^, ^11^, ^12^ The notion that trust is built over time and must continuously be monitored and re‐evaluated, is underscored throughout the PHR and social network literature,^1^, ^8^, ^10^, ^13^ but remains understudied.^1^, ^10^, ^13^ With the recognition that trust is a dynamic, socially embedded process and extends beyond a simplified view as a variable, it requires a methodology that reflects this.^10^

**Figure 1:**
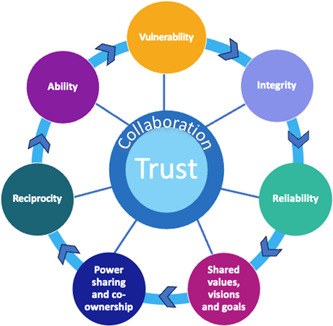
A multidimensional view of trust. The figure displays the seven dimensions of trust as identified in the scoping review by Gilfoyle et al.^16^ Network maps from two of the dimensions, outlined in yellow, were discussed further as prompts in the interviews. The network survey in this study was designed based on these dimensions, with a collaboration question acting as the *name generator* ^18^ for the network survey. The arrows depict the dynamic nature of trust.

PHR partnerships can be viewed as a social network, which is defined as a set of connections among people, organisations or other social actors.^14^ Social network analysis (SNA) provides a set of tools and techniques used to understand these relationships and how these influence behaviour (see File S1 for further information).^14^, ^15^ Gilfoyle et al.,^16^ proposed using SNA to operationalise trust in PHR partnerships, which was explored in their empirical findings.^17^ Specifically, the study^17^ explored seven dimensions of trust between National and Local partners in a PHR project in Ireland called the *PPI Ignite Network* (see setting in Section 2 below). These seven dimensions of trust were informed by a scoping review^16^ and in collaboration with the Research Advisory Group. Each dimension (see Figure 1) had an associated social network question where responses generated a social network map (i.e., a visual representation of the connections among and between people in the PPI Ignite Network). This innovative methodological approach demonstrated by Gilfoyle et al.,^17^ provided insights that were revealed when exploring trust multidimensionally and over time. Findings from this work identified key differences across the trust dimensions over time and by partnership type but lacked the qualitative inquiry necessary to better understand *why* the networks evolved in this way. Indeed, participant experiences, perceptions and beliefs are needed to augment the quantitative findings. This research gap prompted the aim of this qualitative study, *to understand the evolution of trust in the national PPI Ignite Network in Ireland*. Specifically, the objective of this study is to understand contextual factors (i.e., actions or critical events) that influenced the evolution of trust, and why partnership type impacted the evolution of trust.

## 2 METHODOLOGY

This study was granted ethics approval from the University of Limerick Education and Health Sciences Research Ethics Committee (#2021_03_16_EHS) and informed consent was received. Utilising a reflexive approach to thematic analysis (described in Section 3.2),^19^ we consulted Braun and Clarkes' ‘15‐point checklist for good *reflexive* TA [thematic analysis]’ as a quality *guide* (not prescription) for this work. We also consulted the ‘Consolidated criteria for reporting qualitative studies (COREQ) 32‐item checklist’, noting that item #22 ‘data saturation’, is not compatible with reflexive thematic analysis.^19^, ^20^ Underscored by Braun and Clarke,^20^ data saturation is seemingly more suitable for other forms of thematic analysis, including more neopositivist approaches like coding reliability thematic analysis.

### 2.1 Theoretical framework and reflexivity

This study is part of a larger study^21^ and is underpinned by the philosophical traditions and language of critical realism.^22^ Specifically, critical realism challenges a positivist worldview, positing that ontology and epistemology are unlinked from each other in that we cannot reduce what the nature of reality is to our knowledge of reality.^23^ Critical realism also challenges the constructivist ontology that reality is *solely* a function of human experience, knowledge and discourse.^23^ Indeed, we fall in the middle of both paradigmatic poles in that although an objective reality exists, we as humans, can only partially come to ‘know’ and understand this deep and vast reality and the mechanisms involved in the production of empirical events.^22^, ^23^, ^24^, ^25^

In line with this worldview, this study explored participants' experience of reality shaped by their context (e.g., cultural, discourse and perceptions), which we interpreted (i.e., a mediated reflection of reality).^19^

Our research team has a breadth of research experience in PHR, migrant health, primary health care, anthropology, and sociology with credentials ranging from PhD student (M. G.), faculty researchers (J. S. and A. M.) and community‐based nongovernmental organization (NGO) staff (Z. H.) All authors are part of the University of Limerick PPI Ignite Network Operational team. Author J. S. is currently involved in the PPI Ignite Network as a Site Lead, while A.M. was the Site Lead for the preceding PPI Ignite@UL grant. Both J. S. and A. M. are PhD supervisors for first author M. G. As a Site Lead, co‐author J. S. was selected for an interview in this study as his voice was an important contribution to the range of input being sought.^26^ This multiplicity of roles (i.e., both researcher, study participant and PhD supervisor) prompted extensive team discussion amongst co‐authors and the Research Advisory Group, where we carefully considered the pros and cons of this multiplicity (see File S2).^26^, ^27^

### 2.2 Setting

#### 2.2.1 PPI ignite network history and structure

PPI in Ireland has been a focal priority for funders and health researchers since 2014.^28^ This priority sparked capacity‐building initiatives, including the launch of the PPI Ignite Award in 2017, where five universities across Ireland were funded as individual *PPI Ignite Teams* by the Irish Health Research Board (HRB) and Irish Research Council (IRC). Expanding on this progress, HRB and IRC funded the national *PPI Ignite Network* (March 2021–2026), ‘aim[ing] to provide a shared voice for PPI across Ireland, aiming to change the research culture, and an important contributor to improving health outcomes for the public’.^29^

The PPI Ignite Network is comprised of academic, service and community organisations that must collaborate in an efficient, synergistic and cohesive manner to plan, implement and evaluate the PPI initiatives set out by the PPI Ignite Network. Specifically, the PPI Ignite Network contains different ‘types’ of partners, as indicated by its governance structure (see Table 1 below).

**Table 1:** PPI Ignite Network Partner Type and Description.

Partner type	# of partner type in PPI ignite network	Description
Site leads	7	University ‘Lead Sites’ which are the five institutions that previously participated as independent PPI Ignite Teams, plus two new additional institutions who are represented by ‘Site Leads’ (i.e., the local site principal investigators), as well as a national coordinating office, all collectively referred to in this analysis as ‘Site Leads’
National partners	10	National‐level community and health services partners who contribute to national‐level governance and activities called ‘National Partners’
Local partners	39 (at Time 1) and 38 (at Time 2)	Community or health services partners who typically contribute to governance and activities at one university (Lead Site) in the PPI Ignite Network, unless they opt to participate in topical work packages that cut across all Lead Sites.

*Note*: This table presents a summary of partnership type and description in the PPI Ignite Network and the number of partners defined by that partnership type.

Abbreviation: PPI, public and patient involvement.

## 3 SAMPLE AND RECRUITMENT

Following principles of purposive sampling, we invited participants (*n* = 21, seven Site Leads, seven National Partners and seven Local Partners) via email to complete a semistructured interview conducted online via Microsoft Teams if they; (1) completed network surveys at two timepoints; and (2) were deemed key opinion leaders by their peers in each trust dimension for each partnership type (seven Site Leads, seven National Partners and seven Local Partners). Who was deemed a key opinion leader reflects individuals with the top five highest in‐degree centrality scores for each partnership type. In‐degree centrality is a social network measure that calculates the number of incoming connections an individual receives in the network.^14^ People with more incoming connections (i.e., more people agreeing/strongly agreeing with trust statements about them) have a higher in‐degree score. Given the diverse roles and perspectives across the PPI Ignite Network (e.g., Site Leads are exclusively academic partners), inviting seven individuals across each partnership type (i.e., Site Lead, Local Partner and National Partner) helped ensure that we captured diverse opinions. An arrow is shown in the trust dimension network maps^17^ if people agreed or strongly agreed with that statement of trust for that individual. This helped us identify the trust relationships between people in a given network, that is, who trusts whom. Of the 21 individuals invited from the PPI Ignite Network to take part in a semistructured interview, 6 could not be reached. Thus, 15 (four Site Leads, six National Partners and five Local Partners) participated.

### 3.1 Data collection

This study utilised one‐to‐one semistructured interviews (approximately 1 hour in length) conducted (and recorded) virtually via Microsoft Teams. In the interviews, we explored both the participants' context and perceptions of the evolution of trust in the PPI Ignite Network by revieing four social network maps together which were derived from a previous longitudinal study.^21^ In particular, we presented participants with a total of four network maps (two dimensions of trust at two timepoints), which acted as a prompt for discussion. Of the seven dimensions of trust described,^16^ these two dimensions (reliability and power sharing and co‐ownership) were selected because they showed the most observable change in the network maps over time and because it was important to keep the interviews to a reasonable length to not overburden participants.

The social network maps provided a visual representation of the connections among and between people in the PPI Ignite Network. These connections (shown as the arrow in the map) are indicated if people agreed or strongly agreed with that statement of trust for the named individual in the previously administered network survey^17^. To ensure participants were comfortable with this content in the interview, author M. G. developed a short information video (reviewed by one of the Research Advisory Group members), which was provided at least 1 week before the interview date. Author M. G. also provided a ‘refresher’ of the video material at the start of the interviewer via PowerPoint. This refresher included information such as mock network maps and a high‐level overview of how they were constructed. Participants were also given time to ask questions/seek clarification at any point before, during and after the interview. This was all done to help ensure that respondents understood how to interpret the network maps and would be comfortable discussing the data. Throughout the interview process, author M. G. also emphasised that the maps would be used as visual prompts for discussion. The interview guide was informed by Salsberg et al.,^30^ and in collaboration with the Research Advisory Group (see File S3 for the interview guide).

### 3.2 Analytical approach

Following the guiding process outlined by Braun and Clarke,^19^ we used reflexive thematic analysis, to iteratively develop, analyse and interpret our mediated reflection of the data generated from the semistructured interviews. At its core, ‘reflexive thematic analysis captures approaches that fully embrace qualitative research values and the subjective skills the researcher brings to the process’.^31^ ^,^^p.6^ Braun and Clark^31^ ^,p.6^ underscored reflexive thematic analysis as one of ‘a cluster of sometimes conflicting approaches, divergent in procedure and underlying philosophy’ including coding reliability thematic analysis and codebook thematic analysis as described.

We used a reflexive approach to thematic analysis as it is theoretically flexible, embraces the researcher's subjectivity and thus, aligns well with our critical realist worldview.^19^ See File S4 for a detailed description of the reflexive thematic analysis process.

## 4 RESULTS

Following the analytical approach outlined above, three themes were generated based on what people said about their experiences of trust in the PPI Ignite Network and why trust evolved the way it did. These themes describe the evolution of trust as a function of three contextual factors: (1) the set‐up and organisation of the PPI Ignite Network, (2) how people work together and (3) reflection on the process and outcomes. Revealing the nuances within each of these themes, we generated subthemes described in detail below. See File S5 for a table of all themes, subthemes and descriptions. To maintain anonymity, all names are presented as pseudonyms. Institutions and organisations (acting as National or Local Partners) are presented as colours.

### 4.1 Theme 1: The set‐up and organisation of the PPI Ignite Network

This theme is based on participants' descriptions of how the PPI Ignite Network is set‐up and organised based on both internal and external governance processes and how these structures influence people's experience of trust and perception of why it evolved as it did over time. Within the set‐up and organisation of the PPI Ignite Network, there are four subthemes.

#### 4.1.1 Subtheme 1.1: Work packages

This subtheme describes the set‐up and organisation of the PPI Ignite Network and its five ‘work packages’. Each work package had a specific area of focus to progress the work central to the Network's mission (Work package 1—build capacity for PPI in community and academic settings; work package 2—develop accredited education programmes for PPI; work package 3—enhance university policies and procedures to support PPI; work package 4—develop quality improvement and impact and work package 5—create systems for national co‐ordination and functioning.) The work packages were formed within the 1‐year time period of this study and welcomed participation from all partner types (i.e., Lead, Local or National) across the PPI Ignite Network. The work packages were discussed as having implications (both advantageous and disadvantageous) for the evolution of trust as illustrated in the network maps. Specifically, when looking at the network map tapping into the reliability dimension of trust, the work packages were described by a National Partner as beneficial for distributing roles and responsibilities providing opportunities for independence and leadership, as well as opportunities to demonstrate trust, across partnership types:

How it's set up. I think a lot of the work packages are being driven almost independently of the Blue institution, to a certain extent. So, yeah. I think it's good in that sense that you don't have one central hub and everything radiating from it and I think that probably speaks a little bit to the flatter hierarchy within the network and the more even distribution of roles and responsibilities. If you had the Blue institution right in the middle, that would almost imply that everything was being directed through them. (Reese, National Partner)

Comparatively, another National Partner (referring to same network map) discussed feeling on the periphery in the set‐up and execution of the work packages, as they were being driven by the Site Leads. Given the shift to working predominantly in work packages, this was mentioned as disadvantageous for the opportunity to collaborate and be involved in the PPI Ignite Network and therefore be seen as reliable:

I suppose it's harder for me [to say] because we come from the position of a National Partner. There would have been stronger interactions between the Lead Sites because they were developing the work packages and leading on them. Whereas we were there for the steering committee meetings, but we weren't feeding in[to] it at a high level in terms of influence and how the work packages were progressing or strategy. So, it was more sort of a bystander type [of involvement]. (Brooke, National Partner)

The leadership of work packages was further emphasised by a Site Lead, describing the reciprocity and strength of connections depicted in the reliability network maps (where thicker lines denote stronger agreement):

I think that arrow is definitely thicker [stronger rate of agreement] in both directions, you know, and that probably reflects their leadership of work package. (Devon, Site Lead)

Other partner types, including Local Partners and Site Leads, commented on the work package structure influencing the number of connections in the network maps. They noted that the work became more focused with the work packages:

By that stage, we were all quite embedded in our work packages… You know, you were either working on a work package and you were kind of staying within that work package, [and] there wasn't necessarily going to be a huge amount of movement. (Hayden, Local Partner)

#### 4.1.2 Subtheme 1.2: Position/role outside of the PPI Ignite Network

This subtheme describes participants' views about the ways that their roles *outside* of the PPI Ignite Network (i.e., their ‘day job’), might influence a partner's capacity to get involved in the various PPI Ignite Network initiatives including the work packages discussed above. This in turn shaped availability/opportunities for interaction and building of reliability, and power sharing and co‐ownership. For example, when discussing central and less central individuals in the reliability network maps (i.e., the subtle decline in connections), a National Partner said:

A lot of them [National Partners] won't have a person whose sole job it is to think about PPI. So, Kym and Martin, are probably the exceptions in that [PPI] is part of their job remit. But, a lot of the other ones, the smaller circles [in the reliability network] are people who are doing this [as] something on the side. They're involved with the Network, and they see value in it, but it's not part of their [regular] job essentially. And so, the number of hours they're able to commit to doing activities and things like that probably is slightly less. (Reese, National Partner)

A Local Partner further discussed how their involvement was constrained by their job mandate:

…everybody only has a certain amount of time and particularly for me, the PPI Ignite Network is not my core business. It's not [a] core part of my work, it's a very important part of the work, but it's not my core work as a [Local] partner. I don't have the mandate, as much as I would be interested in joining a lot of work packages. I would love the opportunity, and I know I had the opportunity, but I would love to be able to take part in them. But because of the constrictions of my role, I can't, I can really only commit to the core stuff. (Hayden, Local Partner)

For Site Leads, the institutional constraints put in place by their jobs as academics are also highlighted:

…And I appreciate that all of the Site Leads are working academics.

You know, they're not professional staff. I don't think any of them necessarily are released from their day job in order to do this. (Finley, Site Lead)

Thus, participants revealed variations in opportunities to be visible, collaborate and, through that, develop trust with others in the PPI Ignite Network. They revealed that these that variations were shaped by funds and resources outside the PPI Ignite Network that determined availability for core roles and responsibilities within the PPI Ignite Network.

#### 4.1.3 Subtheme 1.3: Availability and distribution of funds and resources within the network

This subtheme describes both the availability and distribution of the network's funds and resources, which are central to the organisation and set‐up of the PPI Ignite Network. One National Partner discussed the challenges and tensions distribution of funds can cause, especially when there is not enough to go around:

…and that the funding of the network, I think led to some challenges around, OK, who? Who's in? Who's out, who gets what money, that that kind of thing.

I mean it [fewer incoming connections] could be down to frustrations with the Blue institution because of all those funding discussions that they had to have. I imagine it was a bit tough because there simply wasn't enough money. (Kym, National Partner)

A Local Partner highlighted the limitations of lack of funding causes:

…particularly for some of the more Community‐based partners you'd be really strict with your time when you're working in that way… If I'm not funded to do something there, I tend to not be able to do it as much. Any of the unfunded work that we do needs to be really key and really critical to our main mandate (Hayden, Local Partner)

#### 4.1.4 Subtheme 1.4: Grant reporting and life cycle

Grant reporting and requirements, describes how the set‐up and organisation of the PPI Ignite Network is influenced by the reporting and requirements dictated by the grant when funds were awarded. For instance, when discussing the decrease in connections in the network maps, a National Partner described the challenges the grant reporting and life cycle can pose on reliability in participatory work:

There is a gap because of the way that the community dynamic works is different than the academic culture. Some of these things in the structure of the grants, like their set timelines, deliverables, and the benchmarks, can't work with the reality of the community and the engagement. (Shae, National Partner)

Additionally, a Site Lead described how the grant reporting and life cycle can create both opportunity and urgency for engagement:

It creates both an opportunity and an urgency to engage with one another around things. It provides deadlines that say you've got to get together and decide what it is you're doing. We've already been convened by the [funding agency] a few months ago to talk about, what would the next phase, phase three, look like? What should we be focused on? So, I think that it's providing the opportunities to get together and the kind of deadlines that say you know, there's going to be a review, that there's going to be a point where people are going to be looking at what you're doing. (Finley, Site Lead)

A Local Partner highlighted the impact the grant cycles have on the quantity and quality of the connections in the network maps, where the quantity may decline, but the agreement rate for reliability of those listed in the network survey might increase:

It's going to follow grant cycles, particularly for collaborations when it's driven by from the academic side. So, a lot of it is going to be ‘I'm working with such and such an organization and another organization on 2 grant applications.’ So, the inbound arrows to them maybe get thicker and collaboration developed, but almost of necessity. (Owen, Local Partner)

### 4.2 Theme 2: How people work together

This theme describes the strategies people have applied individually or collectively to work together given how the Network is set‐up and organised. This includes how people interact with each other and enact their aspirations in the PPI Ignite Network through human agency. Within this overarching theme, we identified five sub‐themes for impacting how people work together and ultimately the evolution of trust.

#### 4.2.1 Subtheme 2.1: Time

This subtheme describes the time that is required to both build and invest in relationships, and the time needed to decide whether to trust another person. This was discussed predominantly by Site Leads and Local Partners. For instance, a Local Partner underscored the need for time to both invest and develop relationships, and whether one has the needed time to invest in them:

It is to do with time to either invest in them and time to yeah, keep developing them, that's. And whether people have time. (Dominique, Local Partner)

A Site Lead highlighted that 1 year, captured in this study, is not enough time to build relationships, and ultimately decide if someone is reliable, hence the little change from Time 1 to Time 2:

I think it takes maybe more than a year like in that year. I don't know how much interaction these different entities had with each other. (Devon, Site Lead)

Participants also mentioned that perhaps, over time, people get to know each other better, and their decision to trust changes because of this. A Site Lead and Local Partner noted why trust declined in the power sharing and co‐ownership network map:

Maybe you start out more trusting at the beginning of relationships. You feel everyone is open to discussion, and you've never had reasons to disagree so you don't realise that there may be disagreements. Whereas maybe as time goes on, and you kind of get stuck into the actual work that's going on, you see how people operate and how open they are and how they react to things. (Robin, Site Lead)

Over the course of a year, I guess you end up building relationships with people and probably know a lot more about what everybody does and maybe how open they are to discussion. (Dominique, Local Partner)

#### 4.2.2 Subtheme 2.2: Frequency and mode of interaction

This subtheme describes how often people were interacting and the mode of which they were interacting (i.e., online or face‐to‐face) and how this influenced the evolution of trust over time. Some participants thought that there was less interaction over time, due to the more focused work after the initial planning stages of the PPI Ignite Network (e.g., through the work packages):

…early on there was more discussion about what the plan is, what are we going to do…trying to bring partners on board whereas time two was maybe focus more on the doing…we have we've agreed the program we need to get on and deliver so there was less opportunities for discussion. (Casey, Local Partner)

Another Local Partner commented on the importance of regular interaction for the reliability dimension of trust:

And the more frequency of your contact with certain people or the more you keep hearing their names as well, you get an idea of, how reliable maybe they are and you're, you're coming into contact with them more and more. (Dominique, Local Partner)

Comparatively, a National Partner commented on the meeting frequency being too much:

I was worried about the amount of time that staff members within the Purple institution were spending attending meetings and what seemed to me to be quite elaborate. (Baker, National Partner)

The mode of meetings, was also mentioned, usually in reference to the impact of COVID‐19 on the evolution of trust in the network maps, such as the need for more face‐to‐face interaction:

It's harder to get to know people when you're just chatting over a screen compared to actually sitting down and having a face‐to‐face interaction. Whenever you're meeting someone face to face, they're all the things like body language, come into play and you know things like that that help you to build a relationship and trust with someone. (Brooke, National Partner)

When discussing the reliability network maps, a Site Lead also commented on the change from online to face‐to‐face interaction, highlighting that this time surveyed was predominantly online interaction:

2021, you know was spent with online interaction … and now that we've returned to face to face, it's just night and day in terms of just getting to know the person and the people, you know. (Devon, Site Lead)

Comparatively, a Local Partner highlighted the benefits of meeting online, such as making it easier to attend Network events, and so forth:

In some ways, COVID made that [attending] slightly easier. Going to an event for something that I had an interest in, that aligned to my work, if it's an hour‐long session on Teams, I can make that work a lot easier than having to take 1/2 or a full day to go to that one‐hour meeting. (Hayden, Local Partner)

#### 4.2.3 Subtheme 2.3: Change in personnel

This subtheme describes how changes in personnel (e.g., staff turnover) impacted how people work together, and ultimately the evolution of trust in the network. Specifically, a Local Partner described the challenges of short‐term contracts impacting the evolution of trust in the network:

But I think that I think the staff turnover things actually it is quite important point because people are volunteers, or many people [that are employed by a given partner in the network] are on precarious contracts short term contracts. (Dominique, Local Partner)

A Site Lead emphasised how a personnel change impacted their connections and the momentum for driving the work forward:

That [change in personnel] would have definitely slowed down the connections and the relationships. We've gotten them back up and running since July, but it's taken the bones of three months really to kind of get around everyone and meet people and discuss concrete actions for the year ahead. And it can take time to kind of get back up again. (Robin, Site Lead)

#### 4.2.4 Subtheme 2.4: Pre‐existing relationships

This subtheme depicts the relationships both within and outside the PPI Ignite Network, through other work or being previously involved in the first PPI Ignite Grant (pre‐dating the current PPI Ignite Network), and how these impact the change in trust over time. One Site Lead described the ‘informal’ network that pre‐existed this grant, enhancing certain connections in the network maps:

…In my opinion, [we had] an informal network prior to the [PPI] Ignite [Network] because five of us and quite a few of our National Partners, already were connected in. We were already networking in an informal way. We had met maybe a couple of times during the three years of the[first] phase one and so, that's probably reflected in here as well. (Devon, Site Lead)

A Local Partner described how seeing an individual outside of the Network through other work helped to form their judgement of them in this Network. Again alluding to the opportunity to judge whether you felt, in this case, they were reliable or not:

When you're talking about his [someone in the networks] actions and behaviors [being] consistent, I'm not only seeing him as part of the PPI network, [but] I'm [also] seeing him doing research on the ground. And the values of PPI run through his work fairly consistently. So, I guess those kind of research partnerships [that] are beyond the network or outside of the network are kind of important [for] helping you make, make some sort of judgment about somebody being dependable or not. (Dominique, Local Partner)

A National Partner described the benefit that certain partners had of being a part of the existing PPI Ignite Grant, and how this opportunity and exposure to each other helped them become more visible in the newly established national Network:

Because they were involved with each other for the first five years, they had a chance to get to know each other and they've collaborated with each other before…. Compared to the likes of ourselves and others coming in at the second time around, and are only new to it, [we] can't contribute as much or don't know people in the same way. (Brooke, National Partner)

#### 4.2.5 Subtheme 2.5: Quality versus quantity of relationships

This subtheme portrays people discussing the quality of relationships improving over time, despite the slight decrease in the number of connections for the two dimensions of trust explored. One Site Lead mentioned this in the following quote:

The fewer arrows here [directed connections in map] could be [because] when you build relationships you kind of focus on the ones that really work. You focus on the people that you work well with. It becomes more about quality rather than quantity. (Robin, Site Lead)

A Local Partner discussed the quality of relationships increasing over time in the following excerpt:

So [I] suppose that kind of looks like quality in some ways, that you're looking at quality versus quantity. And there's a lot of quantity in the first one [Time 1 Network Map], which at the start of a big network, trying to figure out how it all works together, that kind of makes sense. (Hayden, Local Partner)

### 4.3 Theme 3: Reflection on process and outcomes

This theme describes the reflective process of the individual based on their experiences in the Network over time and the importance this kind of appraisal has on the evolution of trust. This would include participants' perceptions of the set‐up and organisation of the PPI Ignite Network and how people work together. This theme extends the previous two themes where participants described their experiences of trust in the PPI Ignite Network and why they think it evolved as it did. In this theme, participants commented on what they think it all means and their perception of this experience (e.g., positive thoughts, critiques, was/was not what they expected). Within this theme, we identified three subthemes.

#### 4.3.1 Subtheme 3.1: Positivity within the network

This theme portrays the general support and positivity participants feel about the PPI Ignite Network, from the work being done and the way it is being executed. This was evident across all partnership types. A National Partner spoke positively about the general leadership (e.g., Site Leads) in the Network:

I think PPI network is doing a good job. I see that the leaders of the network are very serious and they're really into the mission…anytime that I have been participating, I really enjoy it, and I think it gives me that sense that people here are serious. (Shae, National Partner)

Similarly, a Local Partner commented on how being a part of the Network has been a positive experience, being encouraged by others in the Network:

Being part of the network has been absolutely amazing for us because we did set up a PPI panel within Yellow organisation and, [we were] encouraged by Kym in Pink organisation. And having access to the shared learning group is fantastic… (Dominque, Local Partner)

A Site Lead described the resilience of the Network enduring challenges from COVID‐19, and still delivering important initiatives and progressing work:

…despite the challenges we've had, you know, we touched on some of them, particularly COVID and having to interact online, despite those challenges, I think the Network is definitely developing. It's progressing. It's becoming a real sort of entity. I think the festival, it only just happened last month, but I think it was a real success and I think there was a lot of good vibes, good energy coming from that. (Devon, Site Lead)

These positive comments about the PPI Ignite Network underscore participants' optimistic perceptions of the PPI Ignite Network, despite the network maps showing a slight decrease in connections. Specifically, a participant commented on the decline in connections, not as a negative thing, but more as a function of natural engagement. Further, they highlighted that the deepening of certain connections was the important part to focus on. A National Partner mentioned this when the interviewer highlighted the decrease in connections over time in the network maps:

Well, that's happens also because you know I may start with a large team of group of people, but then after getting involved, I may just try to strategically to capitalise on a few that I believe are more reliable or I can better interact with them. So that happens… I think even the quantity decreases. It's important to focus on the quality of the relationship with some of the people that they still are interacting between them. (Shae, National Partner)

A Local Partner expressed similar sentiments, also not surprised by the findings:

No, I think on reflection, it's not hugely surprising…that in some cases the [map line] thickness would go down and, in some others, they'd go up. But generally, the connections would actually fall off. I suppose that's a function of deepening connections with one group almost of necessity entails slackening with others. So, you're going to see fewer arrows. (Owen, Local Partner)

Interestingly, this same Local Partner highlighted that many participants likely selected “neither agree nor disagree” for many connections over time, as some connections deepened, and some slackened over time:

I think it's probably just that it may be, you know, a year later, having met a lot of people in that work, they might have met them since and so they may just feel ‘well, I haven't spoken to that person since, whatever, so it's not going to be a disagree, it'll be a neither agree nor disagree.’ And so, I'd say that's part of it. It's a function of just deepening collaborations and the fall off in others again. (Owen, Local Partner)

A Site Lead reflected on both network maps and also described them as positive in their view:

I mean I think a bit like the reliability domain that you looked at earlier, I think to me it's healthy. It's sort of showing that you know there's a lot of interaction going on in this domain [and] in the reliability domain, hopefully in all of them, that that trust is building. (Devon, Site Lead)

#### 4.3.2 Subtheme 3.2: Motivation and reason for involvement

This subtheme describes participant reflections surrounding motivation, such as why people joined the PPI Ignite Network and the different motivations depending on the type of partnership (i.e., academic vs. charity/NGO, or how contextual factors might affect someone's motivation for involvement) and how this impacted the evolution of trust in the PPI Ignite Network. For example, a National Partner reflected on how context (i.e., relating to the programme of work) can influence peoples' motivation to be involved and, therefore, might be perceived as less reliable:

When you have a program, you need to be mindful and a little bit critical about whether everyone relate[s] to the program. Whether this is something that [is] actually as impactful as everyone envisioned. Is it something that we are doing very well [and/or] something that maybe we have ignored? Those are the context that may increase or decrease someone's motivation to participate that directly and indirectly have an impact on the people, who interact[s] with that person. Like if I became less interested, then I may be perceived as less reliable by someone else. (Shae, National Partner)

A Local Partner reflected on the different motivations in the PPI Ignite Network when discussing the reason for joining the Network and how this might influence the level of power sharing and co‐ownership over time:

…Part of it is the [funding body] have decreed that you have to do this [PPI] and so [academic institutions are] scrambling to join a network because you can't be like you literally can't be outside of it now … you know, there's no point in pretending that's not what's driving a lot of it… That's not the motivation from the non‐academic side… And then there are some researchers who were just really good at this stuff. They have a feel for it. They'd be doing it, something resembling PPI, even if it had no program and no name… And so, I think the motivations vary. (Owen, Local Partner)

#### 4.3.3 Subtheme 3.3: Visibility and opportunity

This last subtheme describes reflections pertaining to the importance of visibility in the PPI Ignite Network, inclusive of the opportunity to be visible, but also an opportunity for critical discussion and opportunity, going forward, to connect with your local partners and to network across the work packages. A Site Lead commented on how the more central people in the Network are those that are more visible in the network:

I would say the bigger bubbles are the visible actors. They're like they're not necessarily the people that I would deal with the closest, but they're the ones who are good at being visible. If that makes sense. I'm not undermining the work that they do at all, but you know they would be, their names are kind of, easily recognizable. (Robin, Site Lead)

This same Site Lead, described the possibility participants confuse visibility with a strong relationship:

Who people see. And I think people think people might confuse because you see someone a lot that you have a strong relationship with them. But actually, what you might actually achieve together or work on together might be very little. (Robin, Site Lead)

The opportunity to be visible was also discussed by some of the partners as an opportunity for trust. For example, a Site Lead commented on how the opportunity to be visible in multiple ways increased the centrality of a specific individual in the resulting trust network maps:

So that means if you are[a] naturally reliable or trustworthy person and you're that exposed to the network, it's going to show up how reliable you are. And at the same time, if you're that exposed to the network and weren't reliable, it would also show up that you were not reliable. But I think the fact that she's that exposed to the network in multiple ways and still came out like, as the largest node. (Simone, Site Lead)

This also touched on the opportunity to lead in the PPI Ignite Network and progress work and thus appear more visible, which was also discussed by a National Partner:

In terms of Site Leads, from my own experience anyway, because they were the ones who were leading on the work packages then they would be maybe thought of as more reliable because they were the ones who were progressing the work. (Brooke, National Partner)

However, the opportunity was discussed from a ‘needs’ perspective in a few ways. A Site Lead commented on the need for the opportunity to have more of a national presence, (e.g., what all of the different sites are doing):

I've heard people kind of say that to me, ‘I thought there'd be more of a national presence as well.’ If I'm honest to say, for example, we had the festival, and I know this doesn't fall in your time points, but we had the national PPI festival there for two weeks in October. And all the events really were all mostly events organised by the Lead Sites. So, I thought that was a really good kind of… It really made it visible the work that each of the Lead Sites are doing. (Robin, Site Leads)

A Local Partner discussed the need for Site Leads to keep linking in with their Local Partners, and establishing more face‐to‐face connections:

I think that to me would highlight the need to for the Local leads [i.e., Site Leads] to keep linking in with their [Local Partners] and maybe looking to have a face‐to‐face meeting or look to keep reinvigorating the [Local Partners], keep the energy within the local network. (Casey, Local Partner)

And then:

Maybe as a learning to some of the Ssite] Leads to kind of keep their [Local] Partners with them. (Casey, Local Partner)

Based on these findings across the three themes, we have presented important questions for reflection (Table 2 below) about the work processes and resource allocation that could be considered for future PHR partnerships.

**Table 2:** Questions for partnership reflections based on themes and subthemes.

Theme	Subtheme	Questions for partnership reflection
Theme 1: The set‐up and organisation of the network	ST 1.1 Work packages	− Who is involved in the set‐up and execution of this structure?
		− Who typically participates?
		− Who is leading the work?
		− Is their collaboration across multiple work packages?
	ST 1.2 Position/role outside of the PPI Ignite Network	− Is the work a part of the persons ‘day job’ and in what capacity?
		− Does the work align with their job mandate outside of the partnership?
		− Does the job outside of partnership limit capacity for involvement?
	ST 1.3 Availability and distribution of funds and resources within the Network	− Are there sufficient resources to adequately fund the work?
		− Who holds and distributes the funds/resources?
		− Is every partner in the network properly resourced?
		− Are grant resources (funding or in‐kind) provided to facilitate the involvement of potentially more marginal partners?
	ST 1.4: Grant reporting and life cycle	− Do the timelines and deliverables of the work align with the community needs and desires?
		− Is the collaboration dictated by the grant cycle deliverables and benchmarks?
Theme 2: How people work together	ST 2.1: Time	− Is there adequate time allotted to build and invest in relationships?
	ST 2.2: Frequency and mode of interaction	− Is there opportunity for face‐to‐face interaction?
		− Is the frequency of interaction suitable for all partners?
	ST 2.3 Change in personnel	− Has staff turnover and its impact been considered?
		− Are there appropriate mitigation strategies in place for change in personnel?
	ST 2.4 Pre‐existing relationships	− Is there a history of working together?
		− Can relationships outside of the partnership be leveraged within this partnership?
		− How can those without pre‐existing relationships be equitably involved in the partnership?
	ST 2.5 Quality vs. quantity of relationships	− Is the strength of relationship improving?
		− Is the number of people collaborating in the Network changing? If so, how?

*Note*: This table highlights themes and subthemes discussed in this paper, presenting questions for reflection about the work and resource allocation that could be considered for future PHR partnerships.

Abbreviation: PHR, participatory health research.

## 5 DISCUSSION

This study builds on previous work conceptualising and operationalising trust in a novel way that embraces context and the dynamic nature of trust.^16^, ^17^ By exploring how participants experienced trust in a PHR partnership, we presented findings that highlight key contextual factors said to influence the evolution of trust over time. These findings were organized by three themes: (1) the set‐up and organisation of the network, (2) how people work together and (3) reflection on the process and outcomes. Overall, participants were very positive about their experiences in the PPI Ignite Network. At the same time, the set‐up and organisation of the network and how people worked together seemingly impacted their ability to be visible in the PPI Ignite Network, which in turn, shaped their availability/opportunities to develop trusting relationships with others in the PPI Ignite Network. This is a key finding of this work. Further, this seemed to vary depending on partnership type with National Partners and Site Leads having more opportunity to demonstrate trust (e.g., via leadership roles or more resources), compared to Local. Other influences on trust in the PPI Ignite Network is described across subthemes such as the mode or frequency of working together, pre‐existing relationships and changes in personnel. We will now explore how these findings compare to other literature, the implications of these findings and the limitations of this study.

Although literature exploring trust temporally in PHR is sparse on this topic, our findings resonate with Armstrong et al's.^10^ exploration of the dynamics of trust drawing on a recently published *longitudinal* PHR project with minority communities in the North East of England from 2014 to 2018. Armstrong et al.,^10^ ^,^^p.13^ also touched on the importance of how people work together, noting the value of the mode of engagement as key for influencing the evolution of trust. For example, they discussed how continuing to share knowledge in an online environment, like emails or phone calls were helpful in maintaining ‘trust at a distance’, but also acknowledged the challenges this created in having difficult dialogue or ‘reconciling issues.^10^’ However, findings from their study and others^32^ mentioned that periodic face‐to‐face interaction was particularly important for maintaining trust between partners.^10^

Interestingly, Armstrong et al.,^10^ also noted the importance of pre‐existing relationships as important for trust. Namely, they described the importance of a shared trust history developed through common experiences and previous encounters. This includes ‘the sharing of time’^10^ ^,p.5^ creating what Schutz and Luckmann^33^ referred to as a ‘we‐relation’, throughout their text titled ‘The Structures of the Life‐World’.^33^ This is a finding also presented in this analysis and social network literature more broadly, where prior relationships are deemed important for shaping future relationships. Indeed, informal relations can circumvent and even replace formal channels established in a network.^32^, ^34^ Thus, it is important to invest time in new partnerships, especially in the early stages, when new environments and cultures are present.^35^ However, this requires substantial time and energy which Lachance et al.,^35^ ^,^^p.522^ discussed as a potential ‘high cost that may outweigh benefits, particularly when those efforts do not work out’ a point mentioned by both community and academic partners.

Other work has also identified that the mismatch between academic calendars as well as community versus academic needs and expectations can pose challenges for working together in partnership,^35^ such as influencing the evolution of trust in a partnership.^10^, ^36^ This was a finding also discussed in our study with a particular emphasis on the ways in which people's day jobs may or may not be in line with their roles in the Network (see ST 1.4). This is a challenge that continues to persist, despite being acknowledged more than 10 years ago.^36^

Armstrong et al.,^10^ and Moore De Peralta^32^ also highlighted challenges with new personnel, specifically a change in staff and volunteers. This can diminish the number of shared experiences between partners compared to those with persistent relationships (i.e., a sustained partnership) (see ST 2.3: change in personnel). This speaks to what observers like Jagosh et al.,^7^ discussed as “trust on trial”, which must be continually negotiated and then renegotiated over time. This temporal element has been discussed as vital throughout the literature, (i.e., withing PHR, SNA^1^, ^8^, ^10^, ^13^ and beyond^37^), despite few studies exploring it in PHR.^7^

Issues of funding and resource distribution is also not a new challenge to PHR, with inherent bias favoring academic and research institutions who typically hold and thus control the distribution of resources.^1^ For instance, existing literature has noted that that mistrust may occur if partners perceived the allocation of funds as unfair or undesirable.^7^, ^10^ This hinders one of the driving principles of PHR, striving for co‐learning and health equity, equalising power between researchers and researched.^38^, ^39^ Wallerstein et al.,^38^ explored how such funding hierarchies perpetuating academic privilege can create barriers for power‐sharing intentions. A study by Oetzel et al.^40^ found that additional stewardship practices (e.g., project advisory boards) in addition to the traditional legal regulations, like institutional review boards (IRBs), can lead to enhanced trust by community partners and promote power sharing between community and academic partners. However, in this study, we found that despite such governance structures existing (e.g., steering committee and public advisory committee) and open for involvement across all partner types, capacity to participate and thus contribute to such structures and be viewed as trusting by others might be constrained by how the PPI Ignite Network is set‐up and organised, including the availability and distribution of funds and resources (ST1.3).^40^

With the differences in distribution of power and resources across partner types (e.g., not everyone having access to funding or having support staff), the *opportunities* to be visible were unequal. This highlighted an important consideration for the possibility of perpetuating inequities throughout the partnership process and, ultimately, its outcomes, despite good intentions of the PPI Ignite Network. Success in partnerships is described, in part, by addressing institutional differences in power and resources which fosters trust in a PHR partnership.^35^ A literature review by Anggraeni et al.,^41^ ^,^^p.364^ found that “the costs and value of participation are inequitably shared between actors.” This issue of potential inequity seemingly impacts trust in PHR.

Indeed this analysis provides an important contribution to the field because, using a social network perspective, we revealed important contextual factors relevant for the evolution of trust in the PPI Ignite Network, while also exploring a specific characteristic of interest in the PPI Ignite Network (i.e., partnership type). Specifically, partnership type seemed to influence an individual's structural position in the Network, also called positional achievement.^34^ This is especially valuable as it allows us to understand who occupies strategic positions in the trust networks, such as who is most central.^34^ As described by Rodrigues^42^ ^,p.2^ “the most central nodes are the most influential ones (pg.2).” Other research has discussed the relevance of additional characteristics including socioeconomic, environmental and cultural factors as potential reasons for differences in trust in the organization.^43^ Although such details were not collected in this study, it is an important consideration for future research.

### 5.1 Implications

This study highlighted important implications for those engaging in PHR partnerships and those who fund such research.

For those who engage in PHR, we found that discussing network maps with partners provided a space for critical dialogue and reflection. This is similar to the advantages of the CBPR conceptual model, where community consultations informing its development promoted self and collective reflection.^44^ This is especially helpful when the partnership is being established. As highlighted by Lachance et al.,^35^ ^,^^pp.521,524^ ‘time on the front end to build capacity and trust creates benefits to the partnership that act to reduce costs of participation over time’. For instance, through these discussions, partners can look at the seven dimensions of trust and identify priority areas for trust in their partnership (i.e., which trust dimensions are most important to their partnership and context).

Second, given that opportunity and visibility to build and maintain trust over time may not be equally available to all partners, it is important to find ways to invest in and commit to equitable relationships as the key to the success (i.e., longevity) of partnerships.^35^ Indeed, this issue of inequity, despite good intentions, can challenge community‐academic partnerships, inhibiting partnership success including synergy and effective long‐term partnerships.^8^, ^35^, ^45^ Thus, it is important for funders and academic institutions to consider this issue and find ways to invest in and commit to equitable relationships as the key to the success of partnerships.^35^ Namely, it is imperative that institutions and funders pay particular attention to and (re)consider the implications of current funding and resource distribution structures. Specifically, the amount of funding and time required to meaningfully do PHR, and how the traditional funding structures (e.g., academics holding and distributing the funds) continue to perpetuate academic privilege^38^ creating barriers for the development and maintenance of trust in PHR partnerships.

### 5.2 Limitations

Findings from this study are limited by the inclusion of a subset of individuals in the PPI Ignite Network, potentially excluding experiences that may align with or differ from those presented here. However, given the breadth of those selected across partnership types, we felt a range of experiences were reflected in this study. It is also important to consider that the interview guide and network surveys informing this study were tailored to suit the PPI Ignite Network and thus may differ when applied to other PHR partnerships. Additionally, as trust requires time to build, looking at trust over 1 year could be limiting.

## 6 CONCLUSION

This qualitative study revealed important contextual factors important for our understanding of the evolution of trust in a national PPI Network in Ireland. Overall, there was a substantial positive reflection on the PPI Ignite Network, but also critical thinking about opportunity and visibility in the PPI Ignite Network, specifically to be viewed as trusting and/or demonstrating trust behaviours. Indeed, the set‐up and organisation of the PPI Ignite Network and how partners work together had important implications for trust equity and should be thoughtfully considered and reflected upon when engaging in and funding PHR.

## AUTHOR CONTRIBUTIONS

All authors have made substantive intellectual contributions to the development of this study. Meghan Gilfoyle conceptualised and led the study, drafted and edited the final manuscript. Jon Salsberg and Anne MacFarlane secured funding. Jon Salsberg, Anne MacFarlane and Zoe Hughes contributed to the conceptualisation of the study, analysis and interpretation of the results, and reviewed and approved the manuscript.

## CONFLICT OF INTEREST STATEMENT

The authors declare no conflict of interest.

## Supporting information

Supporting information.

Supporting information.

Supporting information.

Supporting information.

Supporting information.

## Data Availability

The data that support the findings of this study are available from the corresponding author upon reasonable request.
