# Supplementary material for: Understanding the evolution of trust in a participatory health research partnership: A qualitative study
Source: Health Expect. 2023 Nov 29;27(1):e13918. doi: 10.1111/hex.13918 (PMC10726269; doi:10.1111/hex.13918)
Supplement: Supplementary file 1 — Supporting information. [file HEX-27-e13918-s005.docx]

**Supplementary File 1: Further Descriptions of Social Networks and**

**Social Network Analysis**

**Social Networks**

- A social network can be defined as the set of connections among people, organisations or other social actors (1). In the social sciences, these actors (i.e., nodes) typically represent individuals, groups or organisations (2) whom are connected by a specific type relationship (generating a given social network).
- It is important to emphasise that a social network is a function of the relations (e.g., “who is your friend [*social relation*]”) explored among and between social actors, based on the interest of the researcher (2-4).

**Social Network Analysis**

- Social network analysis (SNA) is described as a distinct perspective and set of techniques used to understand these relationships, how they influence behaviour and how this changes over time (1, 3).
- Social network designs fall into two main categories, whole networks or ego (i.e., node)-centric networks. Studying the whole-network (also known as a *sociometric* approach) examines a set of social actors that are bounded together (analytically) as a social collective (e.g., physicians in a medical practice) (5, 6). Clear boundaries for which social actors are included in the whole-network need to be articulated (6). Ego-centric designs, by contrast, focus on a specific social actor (e.g., a specific physician in a practice) and their relationships in their immediate locality (5, 6). Every ego-centric network resides within a whole-network (5), even if the whole network is never defined. There is also a dyadic level of analysis that looks at characterising network properties between pairs in the network (i.e., two social actors) (5).
- The data collection technique used can be indirect (i.e., observational or based on existing electronic or written records), but is most commonly direct, where data is collected from participants either through surveys or interviews (7, 8). The survey or interview questions often use either a roster or nomination technique(1). With a roster, participants answer questions about people listed, while with nomination, they freely choose individuals (1, 7).
- Specific variables of interest in SNA, generated from network data, are either relational or structural (1). A relational variable is derived from a social actor’s (e.g., survey responder) direct connections (i.e., tie or link). For example, we might be interested in understanding how connected an individual is in the network (i.e., the degree to which they can reach other members in the network) (1). Alternatively, structural (i.e., network-level) variables are derived from all of the connections in the network. For example, understanding how central a person is in the network compared to all others in the network (1). These variables can in turn be explored at both the ego-centric or whole-network level, through a series of measures available to identify, understand and interpret networks of interest (1).
- Specific variables of interest in SNA, generated from network data, are either relational or structural (1). A relational variable is derived from a social actor’s (e.g., survey responder) direct connections (i.e., tie or link). For example, we might be interested in understanding how connected an individual is in the network (i.e., the degree to which they can reach other members in the network) (1). Alternatively, structural (i.e., network-level) variables are derived from all of the connections in the network. For example, understanding how central a person is in the network compared to all others in the network (1). These variables can in turn be explored at both the ego-centric or whole-network level, through a series of measures available to identify, understand and interpret networks of interest (1).
- Which measures are used depends on what we are interested in exploring. For example, “central” people in a network, determined through network measures called centrality (individual/ego level) and centralisation (network-level), has been a critical focus of the network field (1, 9).This focus is due to the prominent role central actors in a network play, occupying what is often deemed an important position in the network (1, 8). Specifically, central actors are thought to have more prestige and visibility in the network, viewed as opinion leaders and are key in the diffusion of ideas and behaviour (1).

**References**

1. Valente TW. Social networks and health: Models, methods, and applications: Oxford University Press; 2010.

2. Brass DJ, Borgatti SP. A brief primer on social network analysis. Social networks at work: Routledge; 2019. p. 1-8.

3. Wasserman S, Faust K. Social network analysis: Methods and applications. 1994.

4. Borgatti SP, Halgin DS. On network theory. Organization science. 2011;22(5):1168-81.

5. Marsden PV. Recent developments in network measurement. Models and methods in social network analysis. 2005;8:30.

6. O’Malley AJ, Marsden PV. The analysis of social networks. Health Services and Outcomes Research Methodology. 2008;8(4):222-69.

7. Scott J. Social Network Analysis. 4th ed. London, United Kingdom: Sage Publications Ltd; 2017. 227 p.

8. Borgatti SP, Everett MG, Johnson JC. Analyzing social networks: Sage; 2018.

9. Borgatti SP, Everett MG. A Graph-theoretic perspective on centrality. Soc Netw. 2006;28(4):466-84.
