# Supplementary material for: Understanding the evolution of trust in a participatory health research partnership: A qualitative study
Source: Health Expect. 2023 Nov 29;27(1):e13918. doi: 10.1111/hex.13918 (PMC10726269; doi:10.1111/hex.13918)
Supplement: Supplementary file 2 — Supporting information. [file HEX-27-e13918-s001.docx]

**Supplementary File 2 - Multiplicity of Roles**

Author JS was PhD supervisor for MG and a study participant. This multiplicity of roles as well as potential for student/supervisor power imbalance, prompted extensive team discussions through multiple virtual team meetings including: 3 meetings amongst supervisors (also co-authors on manuscript) and one meeting with the Advisory Group. During these meetings we recognised and carefully considered the pros and cons of this multiplicity as participant and researcher (1, 2). For example, we considered that while it was essential to include his (JS) perspective and experience to add richness to the content and understanding of context, it might also contribute to power imbalances (e.g., extensive involvement in the research topic). Thus, we strived for reflexivity and transparency about the situation individually (e.g., research diaries) and as a research team (e.g., multiple team discussions talking openly about our positionality and potential power imbalances). Author MG was also mindful of this multiplicity when conducting the interview with JS, which was documented during and after the interview. She also conducted the interview last to ensure the added insight of JS as a researcher for this study and familiarity with SNA did not influence other interviews.

**References**

1. Buys T, Casteleijn D, Heyns T, Untiedt H. A Reflexive Lens on Preparing and Conducting Semi-structured Interviews with Academic Colleagues. Qual Health Res. 2022;32(13):2030-9.

2. Probst B. Both/and: Researcher as participant in qualitative inquiry. Qualitative Research Journal. 2016.
