# Supplementary material for: Understanding the evolution of trust in a participatory health research partnership: A qualitative study
Source: Health Expect. 2023 Nov 29;27(1):e13918. doi: 10.1111/hex.13918 (PMC10726269; doi:10.1111/hex.13918)
Supplement: Supplementary file 3 — Supporting information. [file HEX-27-e13918-s004.pdf]

Information sheet and consent form

Before continuing, please review the information sheet for volunteers by clicking the link below:

LINK: INFORMATION SHEET FOR VOLUNTEERS

EHSREC No: 2021_03_16_EHS

FACULTY OF EDUCATION AND HEALTH SCIENCES
RESEARCH ETHICS COMMITTEE (EHSREC)

Ethical Consent Form

I declare that I am willing to take part in research for the project entitled

"Using a trust lens to inform a social network theory of participatory health research: defining, influencing and measuring the social-relational dimensions of research partnerships"

- I declare that I have been fully briefed on the nature of this study and my role in it and have been given the opportunity to ask questions before agreeing to participate.
- The nature of my participation has been explained to me, and I have full knowledge of how the information collected will be used.
- I am aware that my participation in the interview or focus group will be audio/video recorded and I agree to this. However, should I feel uncomfortable at any time, I can request that the recording software be switched off.
- I am aware that such information may also be used in future academic presentations and publications about this study.
- I fully understand that there is no obligation on me to participate in this study.
- I fully understand that I am free to withdraw my participation without having to explain or give a reason, up to a period of two weeks after the data collection is completed.
- I understand that all other members of the PPI Ignite Network are being invited to participate and that we will be providing opinions on levels of collaboration and trust between partners.
  - Although no one will directly see how I have ranked them, everyone within the study will be able to see the overall network maps and how your organisation was ranked by your peers within it. We realise this may cause discomfort.
- I know that I have been asked not to discuss the content of the focus group discussion, or the identity of its participants with anyone.
- I acknowledge that while the researcher has asked all focus groups participants to maintain confidentiality in the above manner, the researcher cannot guarantee that individual participants will adhere to this request.
- I acknowledge that the researcher does guarantee that they will not use my name or any other information, that would identify me in any outputs of the research.
- I declare that I have read and fully understand the contents of the Research Privacy Notice.
- Consent to Contact about Similar Future Research:
  - I explicitly consent to the University contacting me as part of current or similar future research and holding my contact details on its database for the purpose of contacting me:

○ Yes
○ No

By clicking 'Yes' below, I am providing my consent to participate as indicated above OR click 'No' below, if you choose not to participate at this time.

○ **Yes, I consent to participate**
○ **No, I choose not to participate at this time**

Network Questions Part 1

Thank you for taking part in this study!
You have read the study information letter and have signed an informed consent form, so you understand the purpose of the study and the use to which its findings will be put.
Individuals and institutions will be anonymised in all wider reporting of results.
This questionnaire should take approximately 15 minutes to complete.

Section 1: Network Questions
Level of Collaboration

- For the following questions we are asking you to name organisations you are collaborating with on the PPI Ignite Network.
- When choosing an organisation, think about the specific individuals that represent the organisation in the network.
  - *For example, if you were collaborating with [removed for peer review] choose [remove for peer review].*
- Once you start typing an organisation's **full** name, an auto fill drop-down box will appear. Select the organisation you want. If needed, click here for a full list of network organisations.

Question 1:
a. **Enter up to 7 organisations that you collaborate with on the PPI Ignite Network.**
b. **Beside each of the selected organisations, rank your intensity of collaboration.**
(Please refer to Table A to guide your ranking)

Table A

Level	Definition
(0) No interaction	No interaction, not aware of individual in this organisation
(1) Networking	I am aware of the individual(s), but we have loosely defined roles, little communication, and all decisions made independently
(2) Cooperation	We provide information to each other, have somewhat defined roles, formal communication, but all decisions are made independently
(3) Coordination	We share information and resources, have defined roles, frequent communication, some shared decision making
(4) Coalition	We share ideas and resources, have frequent and prioritised communication, and have a vote in each other's decision making
(5) Collaboration	We belong to one system, our frequent communication is characterized by mutual trust, consensus is reached on all decisions

Intensity of Collaboration

	0 No Interaction	1 Networking	2 Cooperation	3 Coordination	4 Coalition	5 Collaborat
Type organization 1 here:	○	○	○	○	○	○
Type organization 2 here:	○	○	○	○	○	○
Type organization 3 here:	○	○	○	○	○	○
Type organization 4 here:	○	○	○	○	○	○
Type organization 5 here:	○	○	○	○	○	○
Type organization 6 here:	○	○	○	○	○	○
Type organization 7 here:	○	○	○	○	○	○

Network Questions Part 2

Relational questions about 7 PPI Ignite Network Members

- For each of the organisation names (up to 7) you listed in Question 1, please answer the following:

Question 2: On a scale from (1) strongly disagree to (5) strongly agree, please rate the extent to which you agree with the following 7 statements:
**Note: statement 6 has additional descriptions to help guide your selection*

Statement 1:
"I would discuss with [name of network member X] how I honestly feel about my work, negative feelings and frustrations"

	1 Strongly Disagree	2 Disagree	3 Neither agree nor disagree	4 Agree	5 Strongly agree
${q://QID6/ChoiceTextEntryValue/1}	○	○	○	○	○
${q://QID6/ChoiceTextEntryValue/2}	○	○	○	○	○
${q://QID6/ChoiceTextEntryValue/3}	○	○	○	○	○
${q://QID6/ChoiceTextEntryValue/4}	○	○	○	○	○
${q://QID6/ChoiceTextEntryValue/5}	○	○	○	○	○
${q://QID6/ChoiceTextEntryValue/6}	○	○	○	○	○
${q://QID6/ChoiceTextEntryValue/7}	○	○	○	○	○

Statement 2:
"[name of network member X] keeps my interest in mind when making decisions"

	1 Strongly Disagree	2 Disagree	3 Neither agree nor disagree	4 Agree	5 Strongly agree
${q://QID6/ChoiceTextEntryValue/1}	○	○	○	○	○
${q://QID6/ChoiceTextEntryValue/2}	○	○	○	○	○
${q://QID6/ChoiceTextEntryValue/3}	○	○	○	○	○
${q://QID6/ChoiceTextEntryValue/4}	○	○	○	○	○
${q://QID6/ChoiceTextEntryValue/5}	○	○	○	○	○
${q://QID6/ChoiceTextEntryValue/6}	○	○	○	○	○
${q://QID6/ChoiceTextEntryValue/7}	○	○	○	○	○

Statement 3:
"[name of network member X] is dependable. For example, they stick to their word and makes sure their actions and behaviours are consistent

	1 Strongly Disagree	2 Disagree	3 Neither agree nor disagree	4 Agree	5 Strongly agree
${q://QID6/ChoiceTextEntryValue/1}	○	○	○	○	○
${q://QID6/ChoiceTextEntryValue/2}	○	○	○	○	○
${q://QID6/ChoiceTextEntryValue/3}	○	○	○	○	○
${q://QID6/ChoiceTextEntryValue/4}	○	○	○	○	○
${q://QID6/ChoiceTextEntryValue/5}	○	○	○	○	○
${q://QID6/ChoiceTextEntryValue/6}	○	○	○	○	○
${q://QID6/ChoiceTextEntryValue/7}	○	○	○	○	○

Statement 4:
"I am comfortable asking [network member X] to take responsibility for project tasks even when I am not present to oversee what they do"

	1 Strongly Disagree	2 Disagree	3 Neither agree nor disagree	4 Agree	5 Strongly agree
${q://QID6/ChoiceTextEntryValue/1}	○	○	○	○	○
${q://QID6/ChoiceTextEntryValue/2}	○	○	○	○	○
${q://QID6/ChoiceTextEntryValue/3}	○	○	○	○	○
${q://QID6/ChoiceTextEntryValue/4}	○	○	○	○	○
${q://QID6/ChoiceTextEntryValue/5}	○	○	○	○	○
${q://QID6/ChoiceTextEntryValue/6}	○	○	○	○	○
${q://QID6/ChoiceTextEntryValue/7}	○	○	○	○	○

Statement 5:
"I feel that [network member X] shares a vision with PPI Ignite Networks vision and goals?"

	1 Strongly Disagree	2 Disagree	3 Neither agree nor disagree	4 Agree	5 Strongly agree
${q://QID6/ChoiceTextEntryValue/1}	○	○	○	○	○
${q://QID6/ChoiceTextEntryValue/2}	○	○	○	○	○
${q://QID6/ChoiceTextEntryValue/3}	○	○	○	○	○
${q://QID6/ChoiceTextEntryValue/4}	○	○	○	○	○
${q://QID6/ChoiceTextEntryValue/5}	○	○	○	○	○
${q://QID6/ChoiceTextEntryValue/6}	○	○	○	○	○
${q://QID6/ChoiceTextEntryValue/7}	○	○	○	○	○

Statement 6:
"I feel that [network member X] is open to discussion* about matters pertaining to the PPI Ignite Network"

**Note: When we say open to discussion, we mean that this individual is willing to engage in frank, open and civil discussion (especially when disagreement exists). The person is willing to consider a variety of viewpoints and talk together (rather than at each other) and you are able to communicate with this individual in an open, trusting manner.*

	1 Strongly Disagree	2 Disagree	3 Neither agree nor disagree	4 Agree	5 Strongly agree
${q://QID6/ChoiceTextEntryValue/1}	○	○	○	○	○
${q://QID6/ChoiceTextEntryValue/2}	○	○	○	○	○
${q://QID6/ChoiceTextEntryValue/3}	○	○	○	○	○
${q://QID6/ChoiceTextEntryValue/4}	○	○	○	○	○
${q://QID6/ChoiceTextEntryValue/5}	○	○	○	○	○
${q://QID6/ChoiceTextEntryValue/6}	○	○	○	○	○
${q://QID6/ChoiceTextEntryValue/7}	○	○	○	○	○

Statement 7:
"I feel that [network member X] trusts me"

	1 Strongly Disagree	2 Disagree	3 Neither agree nor disagree	4 Agree	5 Strongly agree
${q://QID6/ChoiceTextEntryValue/1}	○	○	○	○	○
${q://QID6/ChoiceTextEntryValue/2}	○	○	○	○	○
${q://QID6/ChoiceTextEntryValue/3}	○	○	○	○	○
${q://QID6/ChoiceTextEntryValue/4}	○	○	○	○	○
${q://QID6/ChoiceTextEntryValue/5}	○	○	○	○	○
${q://QID6/ChoiceTextEntryValue/6}	○	○	○	○	○
${q://QID6/ChoiceTextEntryValue/7}	○	○	○	○	○

Framing Questions

Section 2 – Framing Questions
Trust typology

- In this Question, we are interested in learning your views on **the type of trust** you think exists in the PPI Ignite Network at this point in time. The six trust types and their associated definition **are listed below.**

Question 3: Please indicate your views on trust in the PPI Ignite Network at this time. Specifically, what type of trust do you think currently exists in the network?

CLICK ONE OF THE FOLLOWING:

○ **Critical reflexive trust** (Trust that allows for mistakes and where differences can be talked about and resolved)
○ **Proxy trust** (Partners are trusted because someone trusted invited them)
○ **Functional trust** (Partners are working together for a specific purpose and time-frame, but trust may still be present)

- ○ **Neutral trust** (Partners are still getting to know each other there is neither trust nor mistrust)
- ○ **Unearned trust** (Trust is based on member's title or role with limited or no direct interaction)
- ○ **Trust deficit (suspicion or mistrust)** (Partnership members do not trust each other)

PPI preparedness question

Question 4: On a scale from (1) strongly disagree to (5) strongly agree, please rate the extent to which you agree with the following statement:

"I feel my organisation is prepared to support PPI research at this time"

CLICK ONE OF THE FOLLOWING:

1 Strongly disagree	2 Disagree	3 Neither agree nor disagree	4 Agree	5 Strongly agree
○	○	○	○	○

Powered by Qualtrics
