# Supplementary material for: Understanding the evolution of trust in a participatory health research partnership: A qualitative study
Source: Health Expect. 2023 Nov 29;27(1):e13918. doi: 10.1111/hex.13918 (PMC10726269; doi:10.1111/hex.13918)
Supplement: Supplementary file 4 — Supporting information. [file HEX-27-e13918-s003.docx]

Supplementary File 4 - Detailed overview of reflexive thematic analysis process

Author MG kept a reflexive journal throughout the data collection and analysis process to reflect on all assumptions and decisions made throughout. MG became familiarised with the data, by reading (and re-reading) the data, frequently listening back to the video recording of the interview, as well as making note of possible areas of analytical interest. MG also conducted the interviews and transcribed the data verbatim. MG also re-visited fieldnotes and made additional notes about new insights pertaining to the data and context. Using NVivo, MG began the coding process with a deductive orientation, as the posed research questions were influenced by existing study findings (1, 2), while shifting at times to an inductive orientation, embracing the participant experience in their context. MG also shifted from semantic ( “participant-driven, descriptive”(3)(pg.57)) to more latent (“researcher-driven, conceptual” (3)(pg.57)) coding as analytic insights developed throughout the process, and MG could reflect on the conceptual underpinnings of the initial semantic codes (3). We (co-authors MG, JS and AM) coded and refined codes across multiple rounds of discussion. Following, MG searched for patterns of meaning across the codes and generated initial themes. After further discussions as a team with JS and AM, candidate themes were identified. Next, MG refined, defined, and named the themes, which were again discussed with co-authors JS, AM and ZH as well as the PhD Research Advisory Group. After these discussions, MG then revisited the data to refine the candidate themes, going through each transcript one-by-one looking for patterns *within* each of the three themes separately (i.e., reviewed all transcripts for data coded as theme 1 then reviewed all the transcripts for data coded as theme 2 and so on).

**References**

1. Gilfoyle M, Salsberg J, McCarthy M, MacFarlane A, MacCarron P. Exploring the Multidimensionality of Trust in Participatory Health Partnerships-A Network Approach. Frontiers in public health. 2022;10.

2. Gilfoyle M, Salsberg J, MacFarlane A, McCarthy M, MacCarron P. A Longitudinal Exploration of Trust in Participatory Health Research in Ireland - A Network Approach. Under Review. May 22, 2023.

3. Clarke V, Braun V. Thematic analysis: a practical guide. Thematic Analysis. 2021:1-100.
