# Supplementary material for: Understanding the evolution of trust in a participatory health research partnership: A qualitative study
Source: Health Expect. 2023 Nov 29;27(1):e13918. doi: 10.1111/hex.13918 (PMC10726269; doi:10.1111/hex.13918)
Supplement: Supplementary file 5 — Supporting information. [file HEX-27-e13918-s002.docx]

| **Themes and Sub-themes** | | **Description** | **Excerpts** |
| --- | --- | --- | --- |
| **Theme 1** | **The set-up and organisation of partnerships/the network** | **This theme is based on participants’ descriptions of how the PPI Ignite Network is set-up and organized based on both internal and external governance processes and how these structures influence people’s experience of trust and perception of why it evolves over time. Within the set-up and organisation of the network, are several sub-themes (ST) including work packages (ST1.1), position/role outside of the network (ST1.2), availability and distribution of funds and resources (ST1.3), and the grant reporting and requirements (ST1.4).** | |
| ***Sub-theme 1.1*** | ***Work packages*** | This sub-theme describes the set-up and organization of the PPI Ignite Network into the various work packages, each with a specific area of focus to progress the work central to the Network’s mission. | *“How it's set up. I think a lot of the work packages are being driven almost independently of the Blue institution, to a certain extent. So, yeah. I think it's good in that sense that you don't have one central hub and everything radiating from it and I think that probably speaks a little bit to the flatter hierarchy within the network and the more even distribution of roles and responsibilities. If you had the Blue institution right in the middle, that would almost imply that everything was being directed through them.” (Reese, National Partner)*  *“I suppose it's harder for me because we come from the position of a national partner to say. There would have been stronger interactions between the lead sites because they were developing the work packages and leading on them. Whereas we were there for the steering committee meetings but we weren't feeding in[to] it at a high level in terms of influence and how the work packages were progressing or strategy. So, it was more sort of a bystander type [of involvement].” (Brooke, National Partner)*  *“I think that arrow is definitely thicker in both directions, you know, and that probably reflects their leadership of work package.” (Devon, Site Lead)*  *“By that stage, we were all quite embedded in our work packages...You know, you were either working on a work package and you were kind of staying within that work package, [and] there wasn't necessarily going to be a huge amount of movement.” (Hayden, Local Partner)* |
| ***Sub-theme 1.2*** | ***Position/role outside of the network*** | This sub-theme describes participants’ views about the ways that their roles *outside* of the PPI Ignite Network, might influence a partner’s capacity to get involved in the various Network initiatives including the work packages discussed above. | *“A lot of them [national partners] won't have a person whose sole job it is to think about PPI. So, Kym and Martin, are probably the exceptions in that [PPI] is part of their job remit. But, a lot of the other ones, the smaller circles [in the reliability network are] people who are doing this is [as] something on the side. They’re involved with the Network, and they see value in it, but it's not part of their [regular] job essentially. And so, the number of hours they're able to commit to doing activities and things like that probably is slightly less.” (Reese, National Partner)*  *“...everybody only has a certain amount of time and particularly for me, the PPI Ignite network is not my core business. It's not [a] core part of my work, it's a very important part of the work, but it's not my core work as a [local] partner. I don't have the mandate, as much as I would be interested in joining a lot of work packages. I would love the opportunity, and I know I had the opportunity, but I would love to be able to take part in them. But because of the constrictions of my role, I can't, I can really only commit to the core stuff.” (Hayden, Local Partner)*  *“...And I appreciate that all of the site leads are working academics. You know, they're not professional staff. I don't think any of them necessarily are released from their day job in order to do this.” (Finley, Site Lead)* |
| ***Sub-theme 1.3*** | ***Availability and distribution of funds and resources*** | This sub-theme describes both the availability and distribution of the Network’s funds and resources, which are central to the organization and set-up of the network. | *“...and that the funding of the network, I think led to some challenges around, OK, who? Who's in? Who's out, who gets what money, umm that that kind of thing. I mean it [fewer incoming connections] could be down to frustrations with the blue institution because of all those funding discussions that they had to have. I imagine it was a bit tough because there simply wasn't enough money.” (Kym, National Partner)*  *“...particularly for some of the more Community-based partners you'd be really strict with your time when you're working in that way... If I'm not funded to do something there, I tend to not be able to do it as much. Any of the unfunded work that we do needs to be really key and really critical to our main mandate.” (Hayden, Local Partner)* |
| ***Sub-theme 1.4*** | ***Grant reporting and life cycle*** | Grant reporting and requirements, describes how the set-up and organization of the network is influenced by the reporting and requirements dictated by the grant when awarded funds. | *“There is a gap because of the way that the community dynamic works is different than the academic culture. Some of these things in the structure of the grants, like their set timelines, deliverables, and the benchmarks, can't work with the reality of the community and the engagement.” (Shae, National Partner)*  *“It creates both an opportunity and an urgency to engage with one another around things. It provides deadlines that say you've got to get together and decide what it is you're doing. We've already been convened by the [funding agency] a few months ago to talk about, what would the next phase, phase three, look like? What should we be focused on? So, I think that it’s providing the opportunities to get together and the kind of deadlines that say you know, there's going to be a review, that there's going to be a point where people are going to be looking at what you're doing.” (Finley, Site Lead)*  *“It's going to follow grant cycles, particularly for collaborations when it's driven by from the academic side. So, a lot of it is going to be ‘I'm working with such and such an organization and another organization on 2 grant applications.’ So, the inbound arrows to them maybe get thicker and collaboration developed, but almost of necessity.” (Owen, Local Partner)* |
| **Theme 2** | **How people work together** | **This theme describes the strategies people have applied individually or collectively to work together given how the Network is set-up and organised. This includes the how people interact with each other and enact their aspirations in the network through human agency. Within this overarching theme, we identified five sub-themes for impacting how people work together and ultimately the evolution of trust. These sub-themes include; time (ST2.1), frequency and mode of interaction (ST2.2), change in personnel (ST 2.3), pre-existing relationships (ST 2.4), and quality vs quantity of relationships (ST2.5).** | |
| ***Sub-theme 2.1*** | ***Time*** | This sub-theme describes the time that is required to both build and invest in relationships, and the time needed to decide to trust another person. This was discussed predominantly by site leads and local partners. | *“It is to do with time to either invest in them and time to yeah, keep developing them, that's. And whether people have time.” (Dominique, Local Partner)*  *“I think it takes maybe more than a year like in that year. I don't know how much interaction these different entities had* *with each other.”*  *(Devon, Site Lead)*  *“Maybe you start out more trusting at the beginning of relationships. You feel everyone is open to discussion, and you've never had reasons to disagree so you don't realise that there may be disagreements. Whereas maybe as time goes on, and you kind of get stuck into the actual work that's going on, you see how people operate and how open they are and how they react to things.” (Robin, Site Lead)*  *“Over the course of a year, I guess you end up building relationships with people and probably know a lot more about what everybody does and maybe how open they are to discussion.” (Dominique, Local Partner)* |
| ***Sub-theme 2.2*** | ***Frequency and mode of interaction*** | This sub-theme describes how often people were interacting and the mode of which they were interacting (i.e., online or face-to-face) and how this influenced the evolution of trust over time. | *“...early on there was more discussion about what the plan is, what are we going to do...trying to bring partners on board whereas time two was maybe focus more on the doing...we have we've agreed the program we need to get on and deliver so there was less opportunities for discussion.” (Casey, Local Partner)*  *“And the more frequency of your contact with certain people or the more you keep hearing their names as well, you get an idea of, how reliable maybe they are and you're, you're coming into contact with the more and more.” (Dominique, Local Partner)*  *“I was worried about the amount of time that staff members within the Purple institution were spending attending meetings and what seemed to me to be quite elaborate.” (Baker, National Partner)*  *“It's harder to get to know people when you're just chatting over a screen compared to actually sitting down and having a face-to-face interaction. Whenever you're meeting someone face to face, they're all the things like body language, come into play and you know things like that that help you to build a relationship and trust with someone.” (Brooke, National Partner)*  *“2021, you know was spent with online interaction...and now that we've returned to face to face, it's just night and day in terms of just getting to know the person and the people, you know.” (Devon, Site Lead)*  *“In some ways, COVID made that [attending] slightly easier. Going to an event for something that I had an interest in, that aligned to my work, if it's an hour-long session on Teams, I can make that work a lot easier than having to take 1/2 or a full day to go to that one-hour meeting.” (Hayden, Local Partner)* |
| ***Sub-theme 2.3*** | ***Change in personnel*** | This sub-theme describes how change in personnel (i.e., staff turnover) impacted how people work together, and ultimately the evolution of trust in the network. | *“But I think that I think the staff turnover things actually it is quite important point because people are volunteers, or many people [that are employed by a given partner in the network] are on precarious contracts short term contracts.” (Dominique, Local Partner)*  *“That [change in personnel] would have definitely slowed down the connections and the relationships. We've gotten them back up and running since July, but it's taken the bones of three months really to kind of get around everyone and meet people and discuss concrete actions for the year ahead. And it can take time to kind of get back up again” (Robin, Site Lead)* |
| ***Sub-theme 2.4*** | ***Pre-existing relationships*** | This sub-theme describes the relationships both within and outside the Network, through other work or being previously involved in the first PPI Ignite Grant (pre-dating the National Network), and how these impact the change in trust over time. | *“…In my opinion, [we had] an informal network prior to the [PPI] Ignite [Network] because five of us and quite a few of our national partners, already were connected in. We were already networking in an informal way. We had met maybe a couple of times during the three years of the[first] phase one and so, that's probably reflected in here as well.” (Devon, Site Lead)*  *“When you're talking about his [someone in the networks] actions and behaviors [being] consistent, I’m not only seeing him as part of the PPI network, [but] I’m [also] seeing him doing research on the ground. And the values of PPI run through his work fairly consistently. So, I guess those kind of research partnerships [that] are beyond the network or outside of the network are kind of important [for] helping you make, make some sort of judgment about somebody being dependable or not.” (Dominique, Local Partner)*  *“Because they were involved with each other for the first five years, they had a chance to get to know each other and they've collaborated with each other before. ...Compared to the likes of ourselves and others coming in at the second time around, and are only new to it, [we] can’t contribute as much or don't know people in the same way.” (Brooke, National Partner)* |
| ***Sub-theme 2.5*** | ***Quality vs quantity of relationships*** | This sub-theme describes people discussing the quality of relationships improving over time, despite the slight decrease in the number of connections for the two dimensions of trust explored. | *“The fewer arrows here could be [because] when you build relationships you kind of focus on the ones that really work. You focus on the people that you work well with. It becomes more about quality rather than quantity.” (Robin, Site Lead)*  *“So [I] suppose that kind of looks like quality in some ways, that you're looking at quality versus quantity. And there's a lot of quantity in the first one [Time 1 Network Map], which at the start of a big network, trying to figure out how it all works together, that kind of makes sense. (Hayden, Local Partner)* |
| **Theme 3** | **Reflection on process and outcomes** | **This theme describes the reflective process of the individual based on their experiences in the Network over time and the importance this kind of appraisal has on the evolution of trust. This would include participant’s perceptions of set-up and organization of the network and how people work together. This theme extends the previous two themes where participants described their experiences of trust in the network and why they think it evolved as it did, and now comment on what they think it all means and their perception of this experience (i.e., positive thoughts, critiques, was/ was not what they expected). Within this theme, we identified three sub-themes from the data, including: 1) positivity within the network (ST3.1), 2) motivation and reason for involvement (ST 3.2), and 3) visibility and opportunity (ST 3.3).** | |
| ***Sub-theme 3.1*** | ***Positivity within the PPI Ignite Network*** | This theme describes the general support and positivity participants feel about the network, from the work being done and the way it is being executed. | *“I think PPI network is doing a good job. I see that the leaders of the network are very serious and they're really into the mission […..] anytime that I have been participating, I really enjoy it, and I think it gives me that sense that people here are serious.” (Shae, National Partner)*  *“Being part of the network has been absolutely amazing for us because we did set up a PPI panel within Yellow organisation and, [we were] encouraged by Kym in Pink organization. And having access to the shared learning group is fantastic...” (Dominque, Local Partner)*  *“...despite the challenges we've had, you know, we touched on some of them, particularly COVID and having to interact online, despite those challenges, I think the network is definitely developing. It's progressing. It's becoming a real sort of entity. I think the festival, it only just happened last month, but I think it was a real success and I think there was a lot of good vibes, good energy coming from that.” (Devon, Site Lead)*  *“Well, that's happens also because you know I may start with a large team of group of people, but then after getting involved, I may just try to strategically to capitalize on a few that I believe are more reliable or I can better interact with them. So that happens ... I think even the quantity decreases. It's important to focus on the quality of the relationship with some of the people that they still are interacting between them.” (Shae, National Partner)*  *“No, I think on reflection, it's not hugely surprising ...that in some cases the [map line] thickness would go down and in some others, they'd go up. But generally the connections would actually fall off. I suppose that's a function of deepening connections with one group almost of necessity entails slackening with others. So you're going to see fewer arrows.” (Owen, Local Partner)*  *“I think it's probably just that it may be, you know, a year later, having met a lot of people in that work, they might have met them since and so they may just feel ‘well, I haven't spoken to that person since, whatever, so it's not going to be a disagree, It'll be a neither agree nor disagree.’ And so I'd say that's part of it. It's a function of just deepening collaborations and the fall off in others again.” (Owen, Local Partner)*  *“I mean I think a bit like the reliability domain that you looked at earlier, I think to me it's healthy. It's sort of showing that you know there's a lot of interaction going on in this domain [and] in the reliability domain, hopefully in all of them, that that trust is building.” (Devon, Site Lead)* |
| ***Sub-theme 3.2*** | ***Motivation and reason for involvement*** | This sub-theme describes participant reflections surrounding motivation, such as why people joined the network and the different motivations depending on the type of partnership (i.e., academic vs charity/NGO, or how contextual factors might affect someone’s motivation for involvement) and how this impacted the evolution of trust in the Network. | *“When you have a program, you need to be mindful and a little bit critical about whether everyone relate[s] to the program. Whether this is something that [is] actually as impactful as everyone envisioned. Is it something that we are doing very well [and/or] something that maybe we have ignored? Those are the context that may increase or decrease someone's motivation to participate that directly and indirectly have an impact on the people, who interact[s] with that person. Like if I became less interested, then I may be perceived as less reliable by someone else.” (Shae, National Partner)*  *“...Part of it is the [funding body] have decreed that you have to do this [PPI] and so [academic institutions are] scrambling to join a network because you can't be like you literally can't be outside of it now...you know, there's no point in pretending that's not what's driving a lot of it...That's not the motivation from the non-academic side...And then there are some researchers who were just really good at this stuff. They have a feel for it. They'd be doing it, something resembling PPI, even if it had no program and no name...And so, I think the motivations vary.”* *(Owen, Local Partner)* |
| ***Sub-theme 3.3*** | ***Visibility and opportunity*** | This last sub-theme describes reflections pertaining to the importance of visibility in the network, inclusive of the opportunity to be visible, but also opportunity for critical discussion and opportunity, going forward, to connect with your local partners and network across the work packages. | *“I would say the bigger bubbles are the visible actors. They're like they're not necessarily the people that I would deal with the closest, but they're the ones who are good at being visible. If that makes sense. I'm not undermining the work that they do at all, but you know they would be, their names are kind of, easily recognizable.” (Robin, Site Lead)*  *“Who People see. And I think people think people might confuse because you see someone a lot that you have a strong relationship with them. But actually, what you might actually achieve together or work on together might be very little.” (Robin, Site Lead)*  *“So that means if you are[a] naturally reliable or trustworthy person and you're that exposed to the network, it's going to show up how reliable you are. And at the same time, if you're that exposed to the network and weren't reliable, it would also show up that you were not reliable. But I think the fact that she's that exposed to the network in multiple ways and still came out like, as the largest node.” (Simone, Site Lead)*  *“In terms of site leads, from my own experience anyway, because they were the ones who were leading on the work packages then they would be maybe thought of as more reliable because they were the ones who were progressing the work.” (Brooke, National Partner)*  *‘I've heard people kind of say that to me, ‘I thought there’d be more of a national presence as well.’ If I'm honest to say, for example, we had the festival, and I know this doesn't fall in your time points, but we had the national PPI festival there for two weeks in October. And all the events really were all mostly events organised by the lead sites. So, I thought that was a really good kind of... It really made it visible the work that each of the lead sites are doing.” (Robin, Site Leads)*  *“I think the to me would highlight the need to for the local leads [i.e., site leads] to keep linking in with their [local partners] and maybe looking to have a face-to-face meeting or look to keep reinvigorating the [local partners], keep the energy within the local network.” (Casey, Local Partner)*  *“Maybe as a learning to some of the [site] leads to kind of keep their [local] partners with them.” (Casey, Local Partner)* |
